# Editorial: Multifaceted Cannabinoids: Regulators of Normal and Pathological Function in Metabolic and Endocrine Organs

**DOI:** 10.3389/fendo.2022.848050

**Published:** 2022-02-07

**Authors:** Gergő Szanda, Isabel Gonzalez Mariscal, Tony Jourdan

**Affiliations:** ^1^ Department of Physiology, Faculty of Medicine, Semmelweis University, Budapest, Hungary; ^2^ Instituto de Investigaciones Biomédicas de Málaga, Universidad de Málaga, Málaga, Spain; ^3^ INSERM U1231 Lipides, Nutrition, Cancer (LNC), Dijon, France

**Keywords:** endocannabinoids, CB_1_ receptor degradation, fibrosis, CB_2_ receptor biased signaling, beta cell function, chronic kidney disease, white adipose tissue, pregnancy-parturition

Plant-derived phytocannabinoids, the body’s endogenous cannabinoids (the endocannabinoids) and the cannabinoid receptors are well-recognized modulators of synaptic transmission in the nervous system, and the central effects of cannabinoids have been known to mankind for thousands of years. On the other hand, the physiological and pathophysiological significance and relevance of cannabinoid receptors in peripheral organs have been recognized only recently. Over the past two decades, numerous functions of cannabinoids in peripheral tissues have been documented and it is now generally appreciated that cannabinoids regulate physiological processes in practically all peripheral organs. Moreover, cannabinoids may also contribute to the development of extremely prevalent pathologies such as diabetes and obesity. This characteristic of cannabinoids, together with the increase in recreational and medical cannabinoid usage worldwide, necessitates a clear understanding of cannabinoid actions in metabolic and endocrine organs. The papers in this Research Topic, both original and review articles, wish to spark more interest towards this quickly developing branch of biomedical research.

Deciphering the workings of an entire mediator system requires both basic and clinical research. In this long process, vector-driven expression of receptors in cell lines is often the first step towards understanding receptor function and studying the properties of various ligands. However, overexpression of a receptor has many potential pitfalls and the cannabinoid type 1 receptor (CB_1_R) is a textbook example in this respect. Recombinant CB_1_Rs display high turnover degradation in the proteasomal system and are also prone to couple to non-canonical signalling partners. These processes may well confound experiments if unaccounted for. Now, Horváth et al. provide a solution to these problems – they show that, by tuning down the expression of heterologous CB_1_Rs close to that of endogenous receptors, both the proteasomal degradation and the non-canonical (G_s_ dependent) signalling of CB_1_Rs can be avoided while reaching close-to-physiological receptor levels in the plasma membrane and maintaining canonical signalling. This approach should be considered when studying other G protein coupled receptors (GPCR) in heterologous systems.

Biased coupling of receptors to their signalling partners is a recently recognized phenomenon; one that has potentially huge therapeutic implications. In their current work, Turu et al. demonstrate for the first time that naturally occurring single missense mutations (single-nucleotide polymorphisms) in the cannabinoid type 2 receptor sequence results in biased β-arrestin2 coupling and altered intracellular trafficking. Both mutations are associated with certain psychiatric conditions, raising the possibility that single-nucleotide polymorphisms of the CB_2_R, or other GPCRs for that matter, may lead to disease at least partially *via* inducing signalling bias.

Besides biased signalling, dual-acting compounds constitute another new frontier of cannabinoid research. In our Research Topic, Zawatsky et al. demonstrate a new therapeutical use of their third generation peripheral dual-acting CB_1_R antagonist and iNOS inhibitor, MRI-1867. They show that this dual compound, which already proved its therapeutic potential in liver and pulmonary fibrosis (1), also attenuates skin fibrosis in mice. This finding supports the use of MRI-1867 in scleroderma, an autoimmune condition characterized by fibrotic changes that, in 60% of the cases, can progress into pulmonary fibrosis. The possibility that a new wave of such cannabinoid receptor ligands may be used in therapy in the foreseeable future is quite stimulating.

Diabetes is becoming a worldwide public health issue and understanding what drives the pancreatic functions to fail is critical. In recent years, the involvement of the endocannabinoid system in regulating pancreatic function has become more and more accepted. In this Research Topic, Aseer and Egan present an elegant review of the literature in which they summarize data on the presence of key components of the endocannabinoid system within the pancreas and discuss the relevance of the ECS is modulating β-cell function.

Just as diabetes, chronic kidney disease (CKD) also represents a major public health threat with millions of individuals affected worldwide. To date, only a few therapeutic strategies are available and with limited efficacy only. In their review article, Dao and François give a complete overview of CB_1_R involvement in CKD of both metabolic or non-metabolic origin and critically discuss the therapeutic potential of CB_1_R inhibition in the context of this disease.

The white adipose tissue is the hub of lipid metabolism and plays a central effector role in the regulation of intermediary metabolism. It is thus all the more relevant that this peripheral organ possesses every component of a genuine endocannabinoid system. A growing body of evidence shows that endocannabinoids and fat CB_1_Rs together modulate the function of adipocytes both under physiological and pathological conditions. Here, Buch et al. extend our knowledge on adipose tissue endocannabinoids they demonstrate that autocrine activation of CB_1_Rs actually limits fat mobilization from rodent and human adipocytes. This effect may be one of the first steps towards dysregulation and fat mass expansion, hallmarks of obesity and metabolic syndrome.

Similar to the adipose tissue, the presence of a *bona fide* endocannabinoid system in the female genitalia may also open up new clinical possibilities. Elucidating the function of cannabinoids in these reproductive organs is of utmost importance, given that the regulation of the maintenance of pregnancy and the induction and timing of labour is poorly understood. Endocannabinoids, together with steroids, prostanoids and inflammatory cytokines appear to mutually modulate each other’s function in a complex manner to initiate and maintain pregnancy and, finally, to induce parturition. In their review, Kozakiewicz et al. summarize our current knowledge on the interplay between endocannabinoids and these other mediators in the female genitalia and discuss the potential clinical aspects thereof.

Last but not least, we would like to express our gratitude to all of the authors, reviewers and co-editors for their meticulous work on this inspiring Research Topic.

## Author Contributions

The three authors wrote the editorial together and all approved the final version.

## Conflict of Interest

The authors declare that the research was conducted in the absence of any commercial or financial relationships that could be construed as a potential conflict of interest.

## Publisher’s Note

All claims expressed in this article are solely those of the authors and do not necessarily represent those of their affiliated organizations, or those of the publisher, the editors and the reviewers. Any product that may be evaluated in this article, or claim that may be made by its manufacturer, is not guaranteed or endorsed by the publisher.
